# The Serial Effects of Callous‐Unemotional Traits and Gray Matter Density in the Right Dorsomedial Prefrontal Cortex on Social Desirability

**DOI:** 10.1002/pchj.70005

**Published:** 2025-03-03

**Authors:** Rui Li, Ling‐Xiang Xia

**Affiliations:** ^1^ Faculty of Psychology, Beijing Normal University Beijing China; ^2^ Southwest University ‐ Faculty of Psychology Chongqing China

**Keywords:** callous‐unemotional traits, dorsomedial prefrontal cortex, gray matter density, social desirability

## Abstract

Social desirability affects several aspects of human life. However, the neuropsychological mechanisms underlying individual differences in social desirability remain unclear. This study explored the neuroanatomical basis of individual differences in social desirability using regional gray matter density (rGMD) as a brain indicator in a sample of 158 Chinese college students (79 males; *M*_age_ = 21.42, *SD* = 1.96). Next, we tested the serial effects of callous–unemotional traits (a personality inhibitor of social desirability) and the uncovered brain structural correlation on individual differences in social desirability. Our results indicated that rGMD in the right dorsomedial prefrontal cortex (dmPFC) is associated with individual differences in social desirability. Additionally, callous–unemotional traits were negatively associated with individual differences in social desirability through lower rGMD in the right dmPFC. This study provides the serial effects of personality inhibitor and neural correlate on individual differences in social desirability, which facilitates a more complete understanding of social desirability from the perspective of inhibition, and suggests a neuropsychological mechanism underlying lower‐order personality traits.

## 1 Introduction

Social desirability refers to making the self in line with social norms in response to social or normative pressure rather than offering veridical responses (Lanz et al. 2022). It exists in several aspects of social life, including interpersonal relationships, job hunting, clinical practice and social adaptation (Hippel and Trivers 2011). Although it is important to discover the neural mechanisms underlying social desirability, to gain a better understanding of its nature and determinants, only one functional magnetic resonance imaging (MRI) study (Farrow et al. 2015) has reported on the brain correlates of the social desirability response.

Individual differences in social desirability (IDSD) differ from the response of social desirability, which represents a personality factor (Steenkamp et al. 2010) and reflects the frequency or possibility of individuals responding to social or normative pressure across times and situations in daily life. Previous studies have suggested that it is essential to understand the brain mechanisms underlying personality variables (Avinun et al. 2020; Hyatt et al. 2022). Structural brain scans can provide a neuroanatomical index of the neural mechanisms of individual differences in psychological variables (Hyatt et al. 2019), which cannot be achieved with functional scans. Exploring the brain's structural bases of IDSD helps to provide evidence of brain structure to promote understanding of its formation and change.

Voxel‐based morphometry (VBM) is a powerful method for detecting the neuroanatomical correlates of individual differences in psychological characteristics (Cheetham et al. 2014). Regional gray matter density (rGMD) refers to the gray matter distribution within each voxel and is a reliable and favored indicator of brain structural characteristics (Eres et al. 2015). It is a prime candidate biomarker of brain plasticity (Gennatas et al. 2017) and has been widely used to study the neuroanatomical basis of individual differences in personality factors such as dispositional optimism, ambiguity tolerance and achievement motivation (Dandan et al. 2023; Fink et al. 2014; Lai et al. 2020; Wang et al. 2024; Takeuchi et al. 2014).

### 1.1 The Potential Brain Structural Correlates of Individual Differences in Social Desirability

Although literature on the brain correlates of IDSD is lacking, several studies (e.g., Farrow et al. 2015; Jordan et al. 2020) have investigated the neural correlates of the key psychological components of IDSD.

Social desirability includes two dimensions: self‐deception and impression management (Paulhus et al. 2003). Self‐deception is the tendency or response to describe oneself in a favorable way and believe these self‐descriptions unconsciously and honestly, as well as the defensive denial of thoughts and emotions that threaten oneself (Paulhus 1984). It is a kind of positive self‐illusion, referring to a cognitive bias (Hippel and Trivers 2011). Hence, an unconscious cognitive bias toward the self can be considered a key mental component of social desirability.

Impression management refers to the deliberate manipulation of one's public image and purposefully ensuring that others provide good evaluations of oneself. It involves tendencies or responses that exaggerate one's own positive behaviors and conceal one's own improper behaviors (Paulhus 1984), such as a person claiming that they never litter. Impression management relies on the individual's level of self‐control. Therefore, it could be inferred that purposeful self‐control is another basic psychological element of social desirability.

Previous studies (e.g., Farrow et al. 2015; Jordan et al. 2020) suggest that certain prefrontal cortex (PFC) regions, such as the medial prefrontal cortex (mPFC), may be important for both self‐deception (i.e., unconscious cognition bias toward self) and impression management (i.e., purposeful self‐control). For example, a prior study showed that activation of the dorsomedial prefrontal cortex (dmPFC) is related to reading questionnaire items on self‐deception and impression management (*p* < 0.05, family‐wise error [FWE]) under fake good or bad conditions (Farrow et al. 2015). When transcranial magnetic stimulation was applied to the mPFC, overclaiming, which is a type of self‐related deception (Paulhus 1991), was reduced relative to that in the untreated control group (Taylor‐Lillquist et al. 2020). Furthermore, mPFC damage was found to lead to a more honest response regarding the self, suggesting that the mPFC may participate in self‐deception (Jordan et al. 2020).

### 1.2 The Personality Inhibitor of Individual Differences in Social Desirability

Besides neural variables, several other variables, such as school education, emotional intelligence, substance use and social network factors, are believed to be influencing factors of IDSD (Mesmer‐Magnus et al. 2006; Haberecht et al. 2015). Most of these influencing factors are promotive factors of IDSD. Discovering the inhibitors of IDSD could provide a more complete understanding of the mechanisms of social desirability. The personality factor of callous–unemotional (CU) traits seems to be an important inhibitor of IDSD (Laajasalo 2016). CU traits constitute the affective component of psychopathy (Frick et al. 2014), which is characterized by a lack of guilt, empathy and anxiety (Herpers et al. 2012; Veroude et al. 2016). Individuals with high CU traits are selfish, distant and ruthless when exploiting others for their own benefit (Frick and White 2008; Fanti et al. 2017; Romero and Alonso 2017). Increased reward sensitivity and decreased punishment avoidance are the main characteristics of CU (Herpers et al. 2012).

CU traits are important personality inhibitors of IDSD for two reasons. First, the characteristics of CU traits, such as being self‐centered, not caring about their own performance and having a lower impulse inhibition (Herpers et al. 2012) should make individuals less sensitive to social cues (e.g., have difficulty understanding other people's emotions) (Centifanti et al. 2016), which would hinder them from attending to and following social norms. Hence, CU traits would be detrimental to the development of IDSD. Second, CU traits are higher‐order personality traits relative to social desirability. CU traits are more abstract and general traits, referring to more aspects of personal and interpersonal cognition, emotion and attitude, whereas social desirability only focuses on the cognitive distortion of the self in accordance with social norms. From the perspective of the personality hierarchy theory, a higher‐order personality trait can orchestrate the emergence and maturation of lower‐order personality traits (Conley 1984). Therefore, CU traits may influence IDSD.

### 1.3 The Possibly Serial Effects of CU Traits and Certain Common Brain Correlates on Individual Differences in Social Desirability

Few studies (e.g., Leutgeb et al. 2015; Veroude et al. 2016) have explored the brain correlates of CU traits. For example, volumetric reductions and reduced activity in certain PFC regions (e.g., the mPFC, dmPFC and orbitofrontal cortex) have been found in samples of high callous–unemotional adolescents and adults (Bertsch et al. 2013; Leutgeb et al. 2015; Sebastian et al. 2016; Veroude et al. 2016). Compared with adolescents with low CU, the emotional expression process of adolescents with CU is related to weaker ventromedial prefrontal cortex (vmPFC) activation (Marsh et al. 2008). Some of these brain regions (e.g., the mPFC) are pivotal for IDSD. Thus, we hypothesized that some brain correlates would be common between CU traits and social desirability. Specifically, the structural characteristics of certain brain regions (e.g., the mPFC) may be related to both CU traits and social desirability.

Extant literature suggests that higher‐order personality traits may affect the internal developmental trends of brain structures (Kapogiannis et al. 2013; Taki et al. 2013; Ferschmann et al. 2018; Kolla et al. 2022). This effect may occur because systemic experience differences caused by a higher‐order personality trait may modify cortical plasticity over time (Kapogiannis et al. 2013). For instance, regional plastic changes are triggered by persistent personality‐related practices (e.g., Kolla et al. 2022; Owens et al. 2023). CU traits are higher‐order personality traits that can lead to certain long‐term and consistent behaviors in daily life (Mozley et al. 2018). It is plausible that these long‐term behavioral experiences derived from CU traits may lead to changes in the structure of certain PFC regions.

Taken together, we considered that IDSD may be influenced by CU traits and brain structural correlates. We further argued that CU traits and brain structural correlates would have serial effects on IDSD, and that certain common brain structural correlates of CU traits and IDSD might act as mediators thereof. Specifically, CU traits may reduce the gray matter density (GMD) of certain brain regions (e.g., the mPFC), which in turn inhibit the development of IDSD.

### 1.4 The Present Study

We hypothesized that the rGMD in certain PFC regions may mediate the relationship between CU traits and IDSD. Thus, firstly, this study aimed to use VBM with the rGMD to explore the structural correlates of IDSD at the whole brain level. Secondly, we explored the common brain structural correlates of IDSD and CU traits, and the possible serial effect of CU traits and brain structural correlates on IDSD.

## 2 Materials and Methods

### 2.1 Participants

One hundred and fifty‐eight right‐handed college students (79 males; *M*_age_ = 21.42, *SD* = 1.96) from our university in China participated in this study. No participants reported a history of neurological, psychiatric, or substance abuse disorders. All participants completed the questionnaires and underwent structural MRI scans. Informed consent was obtained from all participants before the study. The study adhered to Helsinki Declaration principles and was approved by the Institutional Review Board of Faculty Psychology at Southwest University. Each participant received compensation as an incentive.

### 2.2 Assessment Instruments

#### 2.2.1 Social Desirability

The Balanced Inventory of Desirable Responding (BIDR; Paulhus 1984) with 40 items was utilized for the assessment of IDSD. It contains two subscales: self‐deception (e.g., “I never cover up my mistakes,” reverse scored) and impression management (e.g., “I don't care to know what people think of me”). The participants rated their agreement with the description of their daily behavior based on their true situation on a seven‐point counterbalanced Likert‐type scale, ranging from 1 (*strong disagreement*) to 7 (*strong agreement*). The Chinese version of the BIDR has good reliability and validity (Li and Li 2008). The mean BIDR score was selected as the index of IDSD with higher average scores in the BIDR indicating higher levels of social desirability. Cronbach's alpha coefficient for this measure was 0.818.

#### 2.2.2 Callous–Unemotional Traits

The Inventory of CU Traits (ICU; Frick et al. 2003) was used to assess callous–unemotional traits, using 24 items consisted of three subscales: uncaring (“I work hard on everything I do,” reverse scored), callousness (“The feelings of others are unimportant to me”), unemotional (“I do not show my emotions to others”). The items were assessed on a four‐point Likert‐type scale, ranging from 0 (*not at all true*) to 3 (*definitely true*). The mean ICU scores were used in this study. A higher degree of CU traits corresponded to a higher ICU score. The Chinese version of this scale has been proven to have high reliability and validity (Fang et al. 2020). In this study, 24 items showed high internal consistency (Cronbach's alpha = 0.732).

### 2.3 MRI Data Acquisition and Preprocessing

#### 2.3.1 Structural Scan Acquisition

A 3.0‐T Siemens Trio MRI scanner equipped with a 12‐channel head coil was utilized to acquire a whole‐brain structural MRI sequence. Each participant underwent a single, high‐resolution, three‐dimensional T1‐weighted anatomical image volume acquisition, utilizing the following parameters: field‐of‐view = 256 × 256 mm; repetition time [TR] = 1900 ms; echo time [TE] = 2.52 ms; inversion time (TI) = 900 ms; flip angle = 9°; number of slices = 176; resolution matrix = 256 × 256 mm^2^; slice thickness = 1.0 mm and voxel size = 1 × 1 × 1 mm^3^.

#### 2.3.2 Image Data Preprocessing

Given that diffeomorphic anatomical registration through exponentiated lie algebra (DARTEL) can achieve more accurate localization and sensitivity, all preprocessing steps in the current study were conducted using standard procedures described in the VBM‐DARTEL tutorial within the Statistical Parametric Mapping software 12 (SPM12, Welcome Department of Cognitive Neurology, London, UK; www.fil.ion.ucl.ac.uk/spm/) environment, in the MATLAB R2014a platform (MathWorks Inc., Natick, MA, USA). The procedure involved five stages. Initially, each magnetic resonance image was scrutinized in SPM12 to identify scanner‐induced artifacts or overt anatomical irregularities. Subsequently, to facilitate improved registration, each image was manually reoriented. Third, new segmentation was applied to partition the images into gray matter (GM), cerebrospinal fluid (CSF) and white matter (WM) using the standard unified segmentation model in SPM12. We calculated the affine transformation matrix between each participant's image and the standard template and used the transformation matrix to generate a roughly aligned image for use by DARTEL. The nonlinear deformation field was then used to register the GM and WM images of the participants, and the mutually collated images were then weighted and averaged to obtain the GM and WM templates of the 158 participants. Fourth, the WM and GM templates produced by DARTEL registration were normalized to the Montreal Neurological Institute (MNI) stereotactic space. Finally, all images were smoothed with a Gaussian kernel of 8‐mm full‐width at half‐maximum. The resulting maps representing GMD were used to explore the correlations between brain regions and mean BIDR score.

### 2.4 Statistical Analysis

#### 2.4.1 Gray Matter Density‐Behavior Correlation Analyses

Multiple regression analysis was used to explore the relationship between rGMD and IDSD. Hence, we included the mean BIDR score in the regression model as the variable of interest. External effects of age, sex and total GMD were regressed as covariates. The results were corrected utilizing the non‐stationary cluster correction method (Hayasaka et al. 2004) with a voxel‐wise level of *p* < 0.005 and a cluster level of *p* < 0.01 (Magalhães et al. 2018). All clusters were anatomically defined according to the Anatomical Automatic Labeling (AAL) atlas. We saved the results of the significant clusters as our region of interest (ROI) definition. We then extracted the mean GMD of the ROI using the REX toolbox. The extracted data were used for the subsequent analyzes. For example, Spearman's correlation between the GMD of the ROI and individual differences in callous–unemotional traits was calculated.

#### 2.4.2 Mediation Analysis

We used the PROCESS macro in SPSS 20 (IBM SPSS Inc., Armonk, NY, USA) for mediation analysis. Based on the results of the rGMD–behavior correlation analyzes, we regarded CU traits as the independent variable, social desirability as the dependent variable and rGMD in the right dmPFC as the mediator. The significance of the mediating effect was assessed using a bootstrapping method with 5000 iterations, extracted from the original data to construct 95% bias‐corrected confidence intervals. If the confidence interval did not cover zero, an indirect effect was considered to exist.

## 3 Results

### 3.1 Descriptive Statistics and Correlation

The means, standard deviations and correlation coefficients of the behavioral variables involved in this study are listed in Table 1.

**TABLE 1:** Mean, standard deviation and correlation of IDSD and CU traits (*n* = 158).

	Mean (*SD*)	Range	*r*
IDSD	3.88 (0.61)	2.65–5.58	
CU traits	1.07 (0.25)	0.42–1.67	−0.34**

Abbreviations: CU, callous–unemotional traits; IDSD, individual differences in social desirability; SD, standard deviation.

** *p* < 0.01.

### 3.2 Neural Correlates of Individual Differences in Social Desirability and CU Traits

**FIGURE 1:**
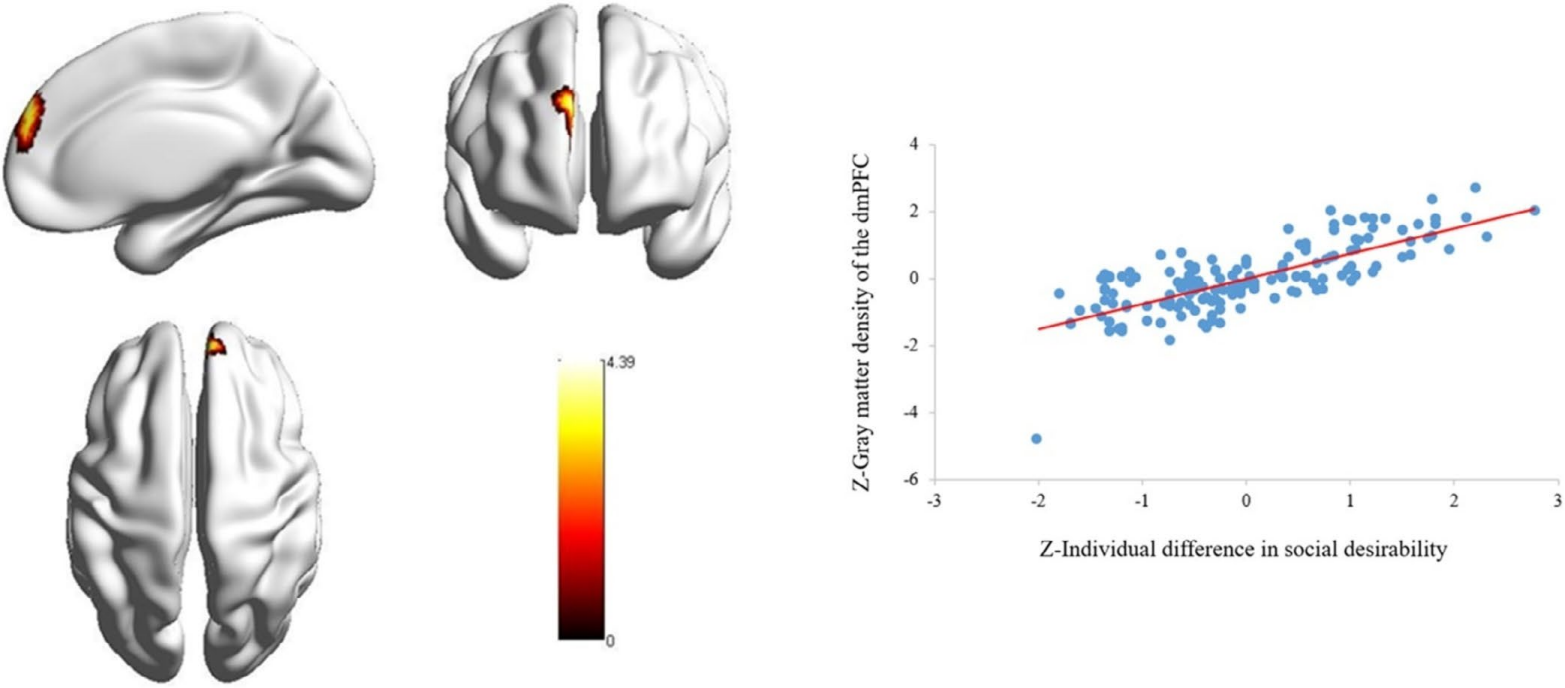
Brain region critical for individual difference in social desirability after controlling for age, sex and global gray matter density (GMD). (a) Individual difference in social desirability was positively associated with the regional GMD (rGMD) in the right dorsomedial prefrontal cortex (dmPFC). (b) Scatter plots showing the association between individual difference in social desirability and the rGMD in the right dmPFC (*r* = 0.757, *p* < 0.001).

To determine the neuroanatomical correlates of IDSD, we examined the relationship between IDSD and GMD at the whole‐brain level. After controlling for age, sex and global GMD and setting IDSD as an interest variable in a regression model, we found that IDSD was significantly positively correlated with rGMD in the right dmPFC (peak MNI coordinate: *x* = 7.5, *y* = 52.5, *z* = 33; cluster size = 265 voxels; *t* = 4.39, *p* < 0.001) (Figure 1).

Then, the rGMD in the dmPFC was extracted, and the correlation between rGMD in the dmPFC and CU traits was determined. The results showed that the negative correlation between rGMD in the right dmPFC and CU traits was statistically significant (*r* = −0.293, *p* < 0.001).

### 3.3 Mediation Model

**FIGURE 2:**
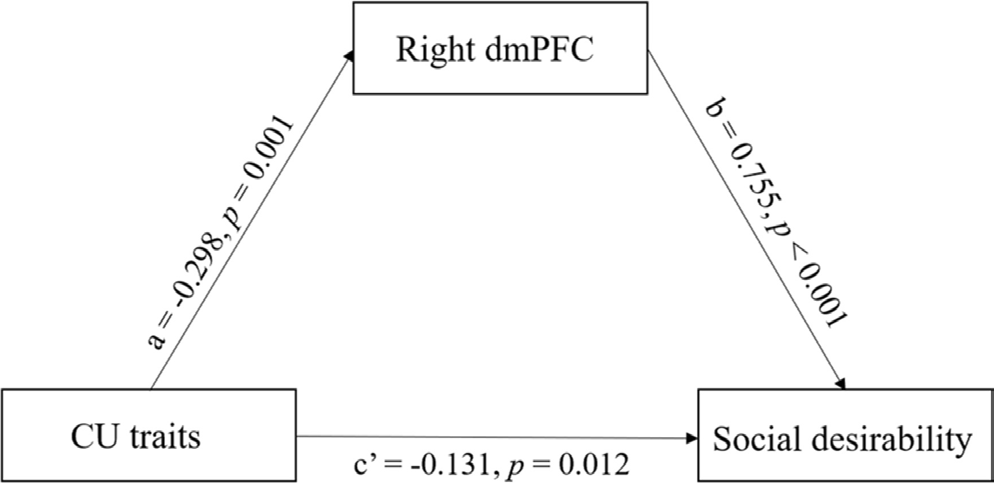
The mediating role of the regional gray matter density (rGMD) of the right dorsomedial prefrontal cortex (dmPFC) in the relationship between Callous–unemotional (CU) traits and social desirability. Social desirability is an individual difference in social desirability.

The mediation model was used to examine whether the rGMD of the right dmPFC acted as a mediator in the relationship between CU traits and IDSD, after controlling for sex, age and global GMD. Bootstrapping analysis indicated that the indirect effect of rGMD on the right dmPFC was statistically significant (*ab* = 0.225, *p = 0*.003, 95% CI = 0.130–0.327). The rGMD of the right dmPFC accounted for 63.201% of the total effect, as shown in Figure 2.

## 4 Discussion

This study investigated the neuropsychological mechanisms underlying IDSD. Specifically, we aimed to uncover the structural correlates of the brain and a potential inhibitor of IDSD as well as the serial effect of CU traits and rGMD of the PFC on IDSD among college students. Whole‐brain correlation analysis showed that higher social desirability scores were linked to greater rGMD in the right dmPFC, which provided new insights into social desirability. Furthermore, we found that CU traits may be associated with IDSD through the rGMD of the dmPFC.

### 4.1 Brain Structural Correlates of Individual Differences in Social Desirability

These results suggest that the dmPFC may be a key brain region for IDSD, which consists of self‐deception (i.e., unconscious cognitive bias toward the self) and impression management (i.e., purposeful self‐control). This finding may clarify a more specific region in mPFC, compared with previous results (Farrow et al. 2015; Jordan et al. 2020; Taylor‐Lillquist et al. 2020).

First, growing evidence (e.g., Casado‐Aranda et al. 2022; Buuren et al. 2020) suggests that the mPFC is a brain structure that is critical for self‐related processing (a psychological basis of social desirability). For example, damage to the mPFC abolishes the self‐reference effect (Philippi et al. 2012). Second, the structural characteristics of the mPFC are important for self‐control (Matsuo et al. 2009). Third, the mPFC is critical for social norm representation (Zinchenko and Arsalidou 2017), and perceiving social norms is a key component of social desirability.

Taken together, existing concepts and findings (Schmidt et al. 2018) suggest that the mPFC is pivotal to self‐cognition, self‐control and social norm representation, which are the psychological bases of social desirability. These studies suggest that the association between the rGMD of the dmPFC and IDSD is reasonable, and that the dmPFC may play an important role in the formation and changes in IDSD.

### 4.2 Relationship Between CU Traits and Individual Differences in Social Desirability

The present results showed that CU traits were negatively associated with IDSD after controlling for age and sex, which is consistent with the results of previous studies (Warren et al. 2015; Laajasalo 2016). CU traits are considered to play a role in predicting IDSD (Laajasalo 2016) because these traits include many antisocial dispositions that make individuals unwilling to develop social desirability. For example, CU traits include less prosocial reasoning, accepting more socially deviant behavior, less impulse inhibition and punishment avoidance, not caring for others, egocentric behavior and a lack of anxiety and guilt (Herpers et al. 2012). These characteristics make people care less about social norms and pressures, and hence do not actively avoid possible punishments for violating social norms. Moreover, these people may not even desire to regard themselves as good people according to traditional social standards.

### 4.3 Brain Structural Correlates of CU Traits

Consistent with the results of previous studies, our results showed that CU traits could negatively predict the rGMD of the dmPFC (e.g., Bertsch et al. 2013; Santana and Aaltonen 2016). First, CU traits are the main elements of psychopathy and seem to be linked to the neuroanatomical features of the mPFC. For example, Santana and Aaltonen (2016) pointed out that, compared to healthy adults, individuals with psychopathy appeared to have decreased rGMD in the mPFC, based on a review of 35 structural MRI articles. Consistently, compared to healthy controls, reductions in the gray matter volume (GMV) in the dmPFC among antisocial offenders with high psychopathic traits were particularly significant (Bertsch et al. 2013; Hirao et al. 2008). Second, CU traits refer to low anxiety, low sensitivity to rewards and a lower likelihood of seeking to avoid punishment (Herpers et al. 2012). The dmPFC is also involved in anxiety, reward and punishment (Martinez‐Horta et al. 2017). Several important components of CU traits are related to structural characteristics of the dmPFC, which may explain the association of CU traits with the rGMD in the dmPFC.

### 4.4 Serial Effects of CU Traits and GMD in dmPFC on Individual Differences in Social Desirability

The results of our mediation model showed that CU traits and the rGMD of the dmPFC had a series of effects on IDSD, and that the rGMD of the dmPFC acted as a mediator of these effects. These results are consistent with the idea in personality hierarchy theory (Conley 1984) and the effect of higher‐order personality traits on brain structures (Kapogiannis et al. 2013; Taki et al. 2013; Ferschmann et al. 2018; Kolla et al. 2022). Our findings suggested that the presence of CU traits, as higher‐order personality factors, impedes the increase in GMD in the right dmPFC, consequently undermining the development of IDSD. These findings offer a novel perspective on the mechanism of social desirability, indicating that the formation and development of IDSD may be due to a series of effects of personality and brain factors, and that certain higher‐order personality factors may influence the formation and development of lower‐order personality factors by altering some structural characteristics of certain brain regions.

### 4.5 Contributions and Limitations

This study makes several contributions to existing literature. First, we have identified a neuroanatomical indicator (i.e., rGMD in the dmPFC) of IDSD. This finding complements existing results regarding the brain correlates of social desirability derived from an fMRI study (Farrow et al. 2015), and it enhances the understanding of the neural mechanism of social desirability. Second, this study facilitates a more complete understanding of social desirability from the perspective of inhibition. Most prior studies focused on the functions of social desirability (Birkeland et al. 2006; Hippel and Trivers 2011). Our study suggests that social desirability may be inhibited by the serial effects of CU traits and rGMD in the dmPFC. Third, our results suggest a potential neuropsychological mechanism for low‐order personalities. Specifically, the formation and changes of low‐order personality traits may be attributed to the serial effects of higher‐order personality and neuroanatomical features. This perspective extends the ideas in personality hierarchy theory (Conley 1984) and enhances our understanding of how higher‐order personality traits shape brain structures (Kapogiannis et al. 2013; Taki et al. 2013; Ferschmann et al. 2018; Kolla et al. 2022).

This study had certain limitations. First, despite the relatively large sample size, all the participants were college students, which may have limited the universal applicability of these findings. Therefore, the study should be replicated among different sample groups (e.g., community adults, clinical patients and delinquent youth). Second, the current results do not support the predictive or causal relationship of the research variables; thus, longitudinal or interventional research (e.g., tDCS, tACS) should be conducted to confirm the causal relationship among the dmPFC, CU traits and IDSD. Third, only the rGMD was considered, and other brain indicators, such as the rGMV, cortical thickness, sulcal depth and some resting‐state functional characteristics, should be studied in the future.

## 5 Conclusions

This study suggested that the rGMD in the right dmPFC is pivotal for IDSD. Furthermore, CU traits and GMD in the right dmPFC had a series of effects on IDSD, and the rGMD in the right dmPFC played a mediating role in the effect. These findings point to a serial effect of personality inhibitor and brain indicator (i.e., rGMD in the dmPFC) on IDSD, which may facilitate a more complete understanding of the mechanism of social desirability from the perspective of inhibition and suggest a neuropsychological mechanism underlying low‐order personality.

## Ethics Statement

This study was approved by the Research Project Ethical Review Committee of the Faculty of Psychology at Southwest University (protocol number H19005), and all the study protocols were in accordance with the standards of the Declaration of Helsinki.

## Consent

All participants signed a written informed consent form before participating in this study.

## Conflicts of Interest

The authors declare no conflicts of interest.
